# Monoclonal antibody induced with inactived EV71-Hn2 virus protects mice against lethal EV71-Hn2 virus infection

**DOI:** 10.1186/1743-422X-7-106

**Published:** 2010-05-26

**Authors:** Guo-hui Chang, Yan-jun Luo, Xiao-yan Wu, Bing-yin Si, Lei Lin, Qing-yu Zhu

**Affiliations:** 1 State Key Laboratory of Pathogen and Biosecurity, Beijing Institute of Microbiology and Epidemiology, 20 Dongda Street, Fengtai District, Beijing, 100071, PR China

## Abstract

**Background:**

Enterovirus 71 (EV71) is a viral pathogen that belongs to the *Picornaviridae *family, EV71-infected children can develop severe neurological complications leading to rapid clinical deterioration and death.

**Results:**

In this study, several monoclonal antibodies (MAbs) were produced by immunizing mice with the inactived EV71 Henan (Hn2) virus strain. The isolated MAbs were characterised by *in vitro *neutralizing analysis and peptide ELISA. ELISA assay showed that the neutralizing monoclonal antibody 4E8 specifically reacted with synthetic peptides which contain amino acid 240-250 and 250-260 of EV71 VP1. The *in vivo *protection assay showed that 4E8 can protect two-day-old BALB/c mice against the lethal challenge of EV71 virus.

**Conclusion:**

The MAb 4E8 could be a promising candidate to be humanized and used for treatment of EV71 infection.

## Background

Enterovirus 71 (EV71) is a viral pathogen within the *Picornaviridae *family and causes clinical diseases in humans with manifestations such as herpangina, aseptic meningitis, encephalitis, pulmonary edema and hand, foot and mouth disease (HFMD). EV71-infected children can develop severe neurological complications that lead to rapid clinical deterioration and death [1]. Since the first instances in 1969 [2], several large epidemics of HFMD have been reported in the Asia-Pacific region, especially in Southeast Asia [3-6]. In China, between 1999-2009, HFMD outbreaks caused by EV71 have affected more than 500,000 young children and resulted in more than 200 deaths in cities, such as Beijing, Shenzhen, Guangdong [7-9]. In fact, after the eradication of the poliovirus [10], EV71 has been regarded as the most important neurotropic enterovirus.

Since there is no EV71 vaccine available and treatment is very limited, a humanized monoclonal antibody might be a viable treatment option against EV71 infection in humans. Anti-EV71 MAbs which have specificity and neutralizing activity could be a promising candidate to be humanized and used for treatment of EV71 infection.

EV71 contains a positive-stranded RNA enclosed by capsid proteins VP1, VP2, VP3 and VP4. VP1 is composed of 297 amino acids and has been shown to be immunogenic [11]. It has been reported that the synthetic peptides SP55 and SP70, which contain amino acids 163-177 and 208-222 of VP1, respectively, can elicit neutralizing antibody against EV71 infection [12,13]. Also, immunization using a recombinant VP1 protein of EV71 was shown to confer protection against lethal EV71 infection in newborn mice, indicating that VP1 contains important antigenic sites that contribute to the neutralization of the virus [14,15].

In this study, we generated several MAbs by immunizing mice with purified EV71 virus, strain Henan2 (Hn2). These MAbs were characterised by *in vitro *neutralizing analysis and peptide ELISA. We identified a monoclonal antibody, clone 4E8 with strong neutralizing activity against EV71. The MAb 4E8 specifically reacted with synthetic peptides which containing amino acids 240-250 and 250-260 of VP1 by ELISA assay. The *in vivo *protection test showed 4E8 can partialy protect the mice against the lethal challenge of Hn2 virus.

## Results

### 50% lethal dose (LD_50_) assay

**Figure 1:**
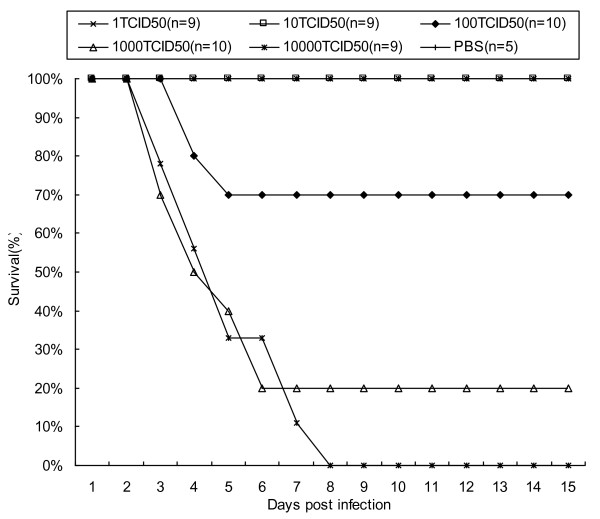
**50% lethal dose (LD_50_) assay**. Two-day-old BALB/c mice were infected intraperitoneally with 100 μl of purified Hn2 virus in dilutions, ranging from 1TCID_50 _to 10000TCID_50_. The survival rates were observed for 21 days.

The EV71 Hn2 strain was isolated from the anal swabs of one HFMD patient from Henan province, P.R.China in 2009. Sequence analysis showed the Hn2 strain was closely related to the EV71 strains detected from the Chinese mainland and grouped into genotype C4 [9]. To determine the 50% lethal viral dosage, two-day-old BALB/c mice were infected intraperitoneally with 100 μl of purified Hn2 virus in dilutions, ranging from 1TCID_50 _(50% tissue culture infectious dose) to 10000TCID_50_. All the mice infected with the 1TCID_50 _and 10TCID_50 _dilutions survived throughout the 21-day observation period, although during the first week post-infection they had a lower average weight than PBS-inoculated mice (data no shown). With an infective dose of the 100TCID_50 _dilution and the 1000TCID_50 _dilution of virus, all of the mice had the typical signs and symptoms of EV71 infection, such as lethargy and paralysis of limbs, within two days post-infection. At the 100TCID_50 _and 1000TCID_50 _dilutions respectively, 70% (7 of 10) and 20% (2 of 10) of the mice survived (Fig.1) and lived throughout the 21-day observation period. With the mice infected with the 10000TCID_50 _dilution of virus, all developed hind-leg paralysis and subsequently died within 9 days post-infection. These results showed that when infected with 100 μl of 300TCID_50 _dilution of stock EV71 Hn2 virus by the intraperitoneal route, 50% of two-day-old BALB/c mice will die within 9 days post-infection.

### Neutralizing antibody assay

After four rounds of panning, seven MAbs were obtained that reacted with high titer in the Hn2 virus antigen-coated ELISA assay. The neutralizing activities of the purified MAbs against EV71 Hn2 virus were analyzed in Vero cells. Two-fold serial dilutions of each MAb were incubated together with 100TCID_50 _Hn2 virus and Vero cells, and the cyto-pathic effects (CPE) of cells was observed 72 h post-infection. A convalescent EV71-infect patient serum was used as a positive control. Among the seven MAbs, three did not have any neutralizing activity, beyond that afforded by a 1:4 dilution of serum from the mock-immunized mice. In contrast, four of the MAbs protected Vero cells from EV71-induced CPE at dilutions ranging from 1:4 to 1:64 (Table 1). The MAbs 4D10 and 3B11 showed a neutralizing activity with complete protection from CPE at dilutions of 1:8 and 1:16, respectively, and the MAbs 4E8 and 4C6 had the same neutralizing titer as the convalescent patient serum, in that they all gave complete protection from EV71-induced CPE at dilutions up to 1:64.

**Table 1:** *In vitro *neutralization assays of monoclonal antibodies*

Antibodies	Neutralization titers
Convalscent patient serum	1:64
4D10	1:8
3B11	1:16
4E8	1:64
4C6	1:64
4G9	<1:4
1A5	<1:4
2F7	<1:4
Normal mouse serum	<1:4

*50 μl of virus suspension containing 100TCID_50 _EV71 Hn2 was pre-incubated with 50 μl of serial two-fold dilutions of antibodies for 30 min before adding to Vero cells in 96-well plates. After 3 days at 37°C, the titer of neutralizing antibody was read as the highest dilution that gave complete protection from CPE.

### Identification of monoclonal antibody epitopes by peptide-coated ELISA

The epitope-specificity of all four neutralizing monoclonal antibodies was tested by peptide-coated ELISA. Synthetic peptides and *E.coli *expressed EV71 VP1 were coated onto the 96-well microtiter plates. Inactived, purified Hn2 virus was used as a positive control. The results showed that all of the neutralizing monoclonal antibodies gave a strong positive signal with inactived Hn2 virus antigen. Among these positive monoclonal antibodies, only 4E8 and 4C6 were strongly reactive with the *E.coli *expressed VP1 protein. MAbs 4E8 and 4C6 also specifically reacted with synthetic VP1 peptides P25 (aa240-250) and P26 (aa250-260)(Table 2). MAb 4C6 showed a weaker reactivity to the synthetic peptides when compared with 4E8.

**Table 2:** Epitope-specificity assay of monoclonal antibody by ELISA*

Antibody	Expressed VP1	Hn2 virus	PBS	Peptide
4D10	0.400	2.551	0.056	-
3B11	0.338	1.740	0.072	-
4E8	1.820	2.665	0.034	1.374 (P25:aa240-250) 1.448 (P26:aa250-260)
4C6	1.727	2.774	0.080	0.705 (P25:aa240-250)
				0.488 (P26:aa250-260)
Normal mouse serum	0.113	0.611	0.045	0.072

*The 96-well microtiter plates were coated with 500 ng of purified EV71 Hn2 virus, *E.coli *expressed VP1 protein or synthetic VP1 peptide P25 or P26. Monoclonal antibodies were at a dilution of 1:2000. OD_450 _nm readings are expressed as the average of of three separate experiments.

### Amino acid sequence analysis of EV71 Hn2 VP1

**Figure 2:**
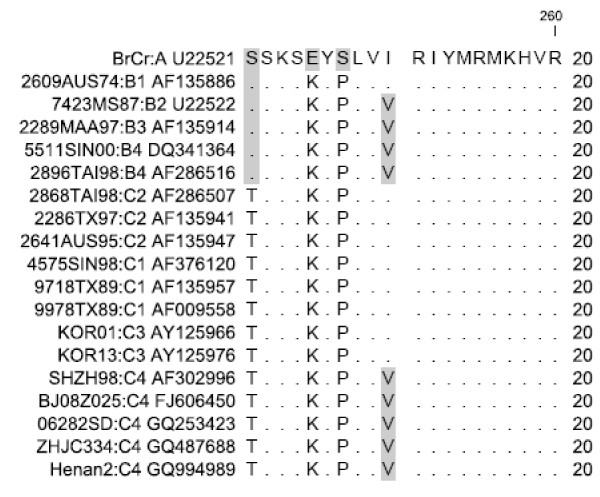
**Sequence alignment of aa240-260 of EV71 VP1 using CLC Main Workbench 5.5 software**. Identical residues with the standard strain BrCr are indicated as dots. Amino acids that differ from the consensus sequence (i.e. the majority of amino acids at a position) are shaded.

The antigenicity of the EV71 Hn2 VP1 protein was determined using the method of Kolaskar and Tongaonkar [16]. Antigenic peptides were predicted based on the occurrence of amino acid residues in experimentally known segmental epitopes. This analysis indicated that there were seven predicted antigenic determinants in the Hn2 VP1 sequence. One of the predicted antigen epitopes (aa242-251: KSKYPLVVRI) was located at the C-terminus of the Hn2 VP1 and corresponded to the synthetic peptide P25 (aa240-250). Sequence alignment indicated that the amino acid sequence of peptide P25 (aa240-250) was less well conserved when compared to the amino acid sequence represented by peptide P26 (aa250-260), which was totally conserved amongst the 19 EV71 strains from sub-genogroups A to C4 (Fig. 2).

### Passive protection test in mice

**Figure 3:**
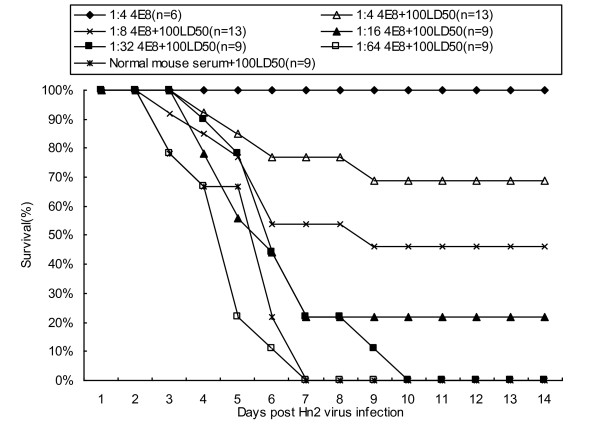
***In vivo *passive protection assay**. Groups of two-day-old BALB/c mice were inoculated with EV71 Hn2 virus (100 LD_50 _per mouse) via the intraperitoneal route. Then, 24 h after infection, they were intraperitoneally administered with serially diluted 4E8 monoclonal antibody. Mice in control groups were administered with normal mice serum, death was monitored until 21 days postinfection.

Two-day-old BALB/c mice were inoculated with the EV71 Hn2 virus at a dose of 100 LD_50 _per mouse by the intraperitoneal (100 μl) route. Then, 24 h later, they were given 100 μl of MAb 4E8 at different dilutions (1:4, 1:8, 1:16, 1:32, 1:64). The results showed that control mice that were administered with 4E8 (1:4) antibody remained healthy and survived throughout the 21-day observation period. The Hn2 inoculated groups that were administered with normal mouse serum had the typical symptoms of EV71 infections by day two post-inoculation and, by day 8 post-inoculation, all the mice had developed hind leg paralysis and subsequently died. In contrast 69% of the Hn2 inoculated mice that were administered with a 1:4 dilution of MAb 4E8 survived throughout the 21-day observation period; The mice administered with 1:8 and 1:16 dilution 4E8 were less protected but, nevertheless 46% and 22%, respectively, survived. Though the inoculated mice administered with a 1:32 dilution 4E8 began to die a little later than mice administered with normal mouse serum, they all died within 10 days. Similar to the inoculated mice administered with normal mouse serum, all of the inoculated mice administered with 1:64 dilution 4E8 died within 7 days (Fig. 3).

## Discussion

The genus *Enterovirus*, belongs to the family *Picornaviridae*, and consists of 66 different subtypes, including polioviruses (PVs), coxsackievirus group A (CVA) and coxsackievirus group B (CVB), echoviruses, and enteroviruses [17,18]. EV-71 was first described in 1969 during an outbreak of HFMD in California that was associated with central nervous system (CNS) complications. EV71 infections are generally mild, such as HFMD and herpangina, but occasionally lead to severe diseases with CNS involvement and clinical symptoms such as aseptic meningitis, poliomyelitis-like paralysis and possibly fatal encephalitis in neonates [19]. Passive transfer of specific antibodies has been shown to reduce the severity of viral infections, including Japanese encephalitis infection [20], varicella infection [21] and coxsackievirus infection [22]. High-quality MAbs which have specificity, avidity and neutralizing activity might be a viable treatment option for EV71 infection in humans.

Previous studies have shown the neutralizing epitope of EV71 was mainly located at the VP1 protein. So we selected synthetic VP1 peptides as the antigen to screen a set of MAbs that we had generated by immunization with inactived EV71 isolated strain Hn2. Among the MAbs we characterised, four were shown to have neutralizing activity, and two can react specifically with the *E.coli *expressed VP1 protein and synthetic peptides of VP1. Experiments are currently in progress to see if the neutralizing MAbs that do not react with VP1 may neutralize the virus by alternative routes, for example, by preventing uncoating of the virus by binding to neighbouring capsid pentamers or by forming aggregates of the viruses as has been observed in other picornaviruses [23,24].

Computerized antigenicity prediction method as used in this study can predict antigenic determinants with about 75% accuracy. Four of the seven epitopes predicted by this method in the EV71 VP1 protein are located at the N-terminus, and only one at the C-terminus. This result is consistent with reports that show the region spanning amino acids 66-132 of VP1 contains the major dimerization domain and cross-reactive enterovirus epitopes [25]. However, there are also reports show that the region between amino acids 132 and 297 is indispensable, and contains important conformational epitope which can react with the convalescent-phase sera [26]. In this study, we have shown that the MAbs 4C6 and 4E8 can specifically react with peptides P25(aa240-250) and P26(aa250-260), one of which overlaps with the predicted C-terminus epitope. Whether the two peptides form a conformational epitope or just a linear neutralizing epitope requires futher research. Nevertheless, it is important that we have shown the 4E8 MAb was able to protect suckling BALB/c mice from a lethal dose of EV71 Hn2 virus, and, therefore, could be a promising candidate to be humanized and used for treatment of EV71 infection.

## Conclusion

In this study, we generated a set of MAbs by immunization BALB/c mice with inactived EV71 isolated strain Hn2. Among the MAbs we characterised, four were shown to have neutralizing activity, and two can react specifically with the *E.coli *expressed VP1 protein and synthetic peptides P25(aa240-250) and P26(aa250-260) of VP1. It is important that we have shown the 4E8 MAb was able to protect suckling BALB/c mice from a lethal dose of EV71 Hn2 virus, and, therefore, could be a promising candidate to be humanized and used for treatment of EV71 infection.

## Materials and methods

### Cells and viruses

Rhabdomyosarcoma cells (RD cell) and Vero cells (derived from African green monkey kidney) were maintained in Dulbecco's modified Eagle's medium (DMEM) containing 10% fetal bovine serum (FBS) plus 2 mM L-glutamine, 100U of penicillin, and 100 μg of streptomycin per ml. The EV71 Hn2 strain was isolated from the anal swabs of one HFMD patient from Henan province, P.R.China in 2009. The patient had the typical clinical symptoms of central nervous system (CNS) involvement, such as fever, exanthemas, aseptic meningitis, and stiffness of the neck [2]. The Hn2 strain was C4 genotype and the complete sequence of Hn2 was submitted to GenBank as accession no. GQ994992.

### Virus purification and titer determination

The isolated Hn2 virus were purified by three rounds of plaque assay in RD cells, then propagated in Vero cells. The infected cells were harvested and completely lysed by three cycles of freeze-thaw. The virus from the tissue culture was purified by precipitation with 7% polyethylene glycol 8000 and then centrifuged onto a 30% sucrose cushion at 25,000 g for 4 h. Virus titers were determined as the median end-point of the tissue culture infectious dose (TCID_50_). Serially diluted virus samples (from 10^-2 ^to 10^-9^) were added to Vero cells in 96-well plates, and eight wells were used at each dilution. The 96-well plates were incubated for 7 days at 37°C, and the TCID_50 _values were measured by determining CPE. The TCID_50 _values were calculated by the method of Reed-Muench [27].

### Determination of LD_50 _in mice

To determine the 50% lethal dose (LD_50_), specific-pathogen-free, two-day-old BALB/c mice were inoculated intraperitoneally with 100 μl of serially diluted Hn2 virus. The survival of the mice was monitored daily, and the LD_50 _was calculated as described by Reed-Muench [27].

### Production of monoclonal antibody

The purified Hn2 virus was inactivated by heating at 56°C for 30 min. Groups of 5 adult (4 weeks old) female BALB/c mice were immunized intraperitoneally in a 50% emulsion of Freund's complete adjuvant with 10 μg heat-inactivated EV71 virus. Two booster doses, again in 50% emulsion with Freund's incomplete adjuvant, were given at 2 weeks intervals. Two weeks after the last immunization, blood samples were obtained from the tail and tested by enzyme-linked immunosorbent assay (ELISA) using purified Hn2 virus as antigen. The fusion of spleen cells with mouse myeloma cell was done as described [28]. For the production of MAbs, BALB/c mice (4-6 weeks old) were injected intraperitoneally with 10^6 ^hybridoma cells per mouse. Ten days later, the ascitic fluid was drained by using an 18-gauge needle. The ascites were centrifuged at 1,000 g for 10 min to remove cells, MAbs were purified from collected supernatants by precipitation with 50% saturated ammonium sulfate (pH 7.0) and dialysised against 0.04M phosphate buffer (pH 6.8). The MAbs were further purified by using protein A agarose columns (Bio-Rad Laboratories).

### *In vitro *neutralizing antibody assay

The presence of neutralizing MAbs was determined by in vitro assay in Vero cell. Monoclonal antibody samples were incubated at 56°C for 30 min to inactivate the complement. Briefly, 50 μl of two-fold antibody dilutions were mixed with equal volumes of 100TCID_50 _of Hn2 virus, and then the mixture was added to monolayers of Vero cells in 96-well plates. All serum samples were tested in 8 wells. After 3 days of growth, the titer of neutralizing antibody was read as the highest dilution that gave complete protection from CPE.

### Peptide ELISA assay

Synthetic peptides spanning the entire sequence of the Hn2 VP1 protein (GenBank accession no. GQ994992) were synthesized by Invitrogen Company (Beijing, P.R.China). Each peptide contains 10 amino acid residues and the levels of antibody reactive to each synthetic peptide were measured by ELISA assay. The 96-well plates were coated overnight at 4°C with 50 μl of 0.1M carbonate buffer (pH 9.6) containing 500 ng of synthetic peptide or *E.coli *expressed EV71 VP1 protein (supplied by Professor Hanzhong Wang, Wuhan Institute of Virology, Chinese Academy of Science). After blocking with 2% bovine serum albumin (BSA), the plates were incubated with 50 μl of MAb at the indicated dilutions at 37°C for 1 h and then washed three times with phosphate buffered saline (PBS) containing 0.1% Tween 20. Horseradish peroxidase-conjugated goat anti-mouse IgG antibody (SantaCruze, USA) was used as secondary antibody at 37°C for 1 h and the reaction was developed by TMB substrate (KPL, Gaithersburg, MD). The absorbance at 450 nm was measured by an ELISA plate reader (Tecan Sunrise, USA). Each assay was performed three times independently.

### Passive protection test in mice

Two-day-old BALB/c mice were obtained from the Laboratory Animal Center, Academy of Military Medical Sciences, Beijing. Institutional guidelines for animal care and use were followed throughout the experiments. Groups of mice were injected intraperitoneally with 100 μl (100LD_50_) of Hn2 virus, followed 24 h later by 100 μl of heat-treated (56°C, 30 min) MAb. Mice in the control group were injected with the same volume of normal mice serum. The mice were monitored for clinical symptoms, paralysis and death up to day 21 post-inoculation.

### Computational analysis of VP1 sequence

Nucleotide sequence and the amino acid sequences were edited and aligned using CLC Main Workbench 5.5 software. The antigenicity of the Hn2 VP1 protein were analyzed using the method of Kolaskar and Tongaonkar [16], and antigen epitopes were predicted using the amino acid sequences of the Hn2 VP1 as query sequences in the Web: http://www.mifoundation.org.

## Competing interests

The authors declare that they have no competing interests.

## Authors' contributions

*GHC *is the principal investigator and draft the manuscript; *YJL, XYW *and *BYS *carried out the the animal test and immunoassays of the MAbs. *LL *participated in the sequence analysis. *QYZ *participated in its design and coordination of the study. All authors read and approved the final manuscript.
